# Composites of Bimetallic Platinum-Cobalt Alloy Nanoparticles and Reduced Graphene Oxide for Electrochemical Determination of Ascorbic Acid, Dopamine, and Uric Acid

**DOI:** 10.1038/s41598-019-48802-0

**Published:** 2019-08-22

**Authors:** Buse Demirkan, Sait Bozkurt, Aysun Şavk, Kemal Cellat, Fulya Gülbağca, Mehmet Salih Nas, Mehmet Hakkı Alma, Fatih Sen

**Affiliations:** 1 Sen Research Group, Department of Biochemistry, Faculty of Arts and Science, Dumlupinar University, Evliya Çelebi Campus, 43100 Kütahya, Turkey; 2 Department of Environmental Engineering, Faculty of Engineering, University of Igdir, Igdir, Turkey

**Keywords:** Biosensors, Hormones

## Abstract

The ultimate aim of this study is to produce a composite of bimetallic platinum-cobalt nanoparticles and reduced graphene oxide (Pt-Co@rGO) based biosensor for the detection of ascorbic acid (AA), dopamine (DA) and uric acid (UA). Those are biologically important molecules with the key functions for the human body. Pt-Co@rGO was synthesized using a microwave-assisted technique and utilized for the production of a highly sensitive and stable electrochemical biosensor. Detailed spectral XPS and Raman analysis, XRD, and TEM/HR-TEM characterization were also studied. Due to the superior activity and excellent conductivity of rGO, well-separated oxidation peaks of these biomolecules is proven by DPV (differential pulse voltammetry) and CV (cyclic voltammetry) measurements. The prepared Pt-Co@rGO-based biosensor showed high electrochemical activity, a broad linear response, high sensitivity, and acceptable limit of detection values for individual and simultaneous determination of AA, DA, and UA, under optimized conditions. The linear range of Pt-Co@rGO was found to be 170–200; 35–1500 and 5–800 µM for AA, DA, and UA, respectively. Moreover, the detection limit of the prepared composite was calculated as 0.345; 0.051; 0.172 µM for AA, DA, and UA, respectively. In the field of electrochemical biosensors, Pt-Co@rGO based sensor is highly promising due to its superior sensitivity and good selectivity properties.

## Introduction

**Figure 1:**
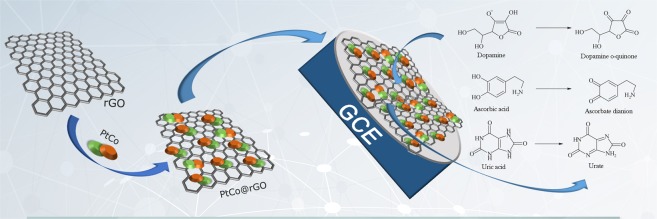
Schematic diagram of the production and detection process of the Pt-Co@rGO biosensor.

AA, UA, and DA are biologically important molecules existing in biofluids (urine and blood) and have significant importance in human metabolism. Abnormal levels of AA (vitamin C), a potent antioxidant from these biomolecules; can lead several illnesses from the common cold to cancer, mental illness, and hepatic diseases. AA is an important component of human nutrition, commonly found in food and beverages. It is also used in animal nutrition, food industry, and cosmetics. Since AA is existing in millimolar levels in the neural system, it is important to improve an easy, fast and sensible method for detection^1,2^. DA is one of the most significant catecholamine neurotransmitters in the neural framework of mammals. In the human body, abnormal levels of DA can cause some diseases such as Huntington’s, Parkinson’s, schizophrenia, etc.^3,4^. UA is an important biological compound found in biological fluids, and it is a product of purine metabolism. UA with abnormal levels indicates the presence of various disorders including Lesch-Nyan disease, gut, celiac, and hyperuricemia^5,6^. The concentration of DA in the extracellular liquid is extremely low in healthy individuals (1 × 10^−8^ to 1 × 10^−6^ M) and even lower levels (1 × 10^−9^ M) are available in Parkinson’s patients^7–9^. Simultaneous detection of these biologically important compounds is a key factor for pathological, clinical, and biological studies. Thus, it is required to develop a rapid, cost-effective, simultaneous detection method with high sensitivity and specificity for these biologically important compounds in clinical trials. However, it is very difficult to achieve electrochemical detection on commercial standard electrodes, due to their high oxidation potential, which causes contamination of the electrode plane. Moreover, when the traditional electrodes were utilized for the detection, oxidation peak potentials of these biomolecules are observing almost in the same region. This cause overlapping of their voltammetric responses and makes very difficult to detect their concentrations using electrochemical methods. AA can be detected using various techniques such as HPLC, electrochemiluminescence, and Raman spectroscopy that also allow the detection of DA and UA, at the same time. However, the high cost and the long operation time of these methods limit their usage^10–12^. In recent studies, it is observed that modified electrodes have advantages over the other techniques, such as low cost, easy to use, fast and wider range of calibration, and considered one of the most promising developments in electrochemistry. The linear responses obtained for the UA are near the upper boundary of the regular range^13,14^. Recently, two-dimensional graphite and a single layer of graphene have attracted attention due to its strong mechanical stability, special plane space, great conductance, and superior electrocatalytic action. Graphite, graphene oxide (GO), and graphene derivatives facilitate the distribution of elements and support catalytically effective metal nanoparticles (NPs) in different catalytic reactions. Thanks to its unique properties such as fast electron transport, biocompatibility; graphene can be applied in all biosensors such as electrochemical, impedimetric, fluorescence biosensors, as well as immunosensors. The electrochemical behaviour of graphene and graphene-based electrochemical sensors (ECS) studies has been extensively conducted in the last few years and reported that graphene will be an important electrode material in electrochemical analyses^15–18^. The layers of GO are covalently encircled by hydroxyl and epoxy groups, and the sides are configured with carboxyl groups. In addition, polymer-modified electrodes have aroused a great deal of interest, due to their porous structure^19–21^. Since the year of 2000, studies have focused on the isolation of graphene and GO derivative nanomaterials. Their two-dimensional structure also allows the presence of localized p-conjugate-electrons on the surface. Furthermore, their high surface/volume ratio makes them an ideal material for electrochemical applications^18,22–24^. For this reason, rGO, which is a GO-based material, was used as supporting material in this work. Many types of nanomaterials were synthesized, and Pt-Co@rGO provided very high performance for the determination of AA, DA, and UA at low potentials, as shown in Table S1 ^25–27^. The conductive matrix of nanocomposite materials is generally formed by Co^27^, Au^28^, Pd^29^, and Pt^30^. As a summary, this study reports that the use of rGO-supported Pt-Co nanoparticles for the simultaneous detection of AA, DA, and UA with low detection limits and wide linear response intervals (Fig. 1). The produced electrochemical biosensor was successfully applied to human serum samples for the improvement of the studies conducting on these biomolecules.

## Experimental

### Materials and apparatus

The majority of the chemicals including graphite powder, cobalt chloride (CoCl_2_), platinum chloride (PtCl_4_), cetyltrimethylammonium bromide (CTAB), hydrogen peroxide (H_2_O_2_, ≥30%) and ammonium persulphate (APS ≥98%), were purchased from Sigma-Aldrich. Distilled water was strained through a Millipore water treatment system (18 MΩ). All glass materials and teflon coated magnetic bars were washed with distilled water. Transmission electron microscope (TEM) observations were carried out using a JEOL 200 kV transmission electron microscope. X-ray diffraction analysis (XRD, Panalitic Emperian Diffractometer) was performed by an Ultima + theta-theta high-resolution goniometer equipped with a Cu-K weld operated at 40 kV and 40 mA. The angular dissolution in 2θ scans was 0.02°. The scanning area was 10° to 90°, and the scanning speed was 4 min^−1^. X-ray photoelectron spectroscopy (XPS) was applied to define the oxidation state of the metals and structural analysis of Pt-Co@rGO. XPS analyses carried out using the Kα bands of Mg as an X-ray source (1253.6 eV, 10 mA). The three-electrode technique was carried out for electrochemical measurements such as cyclic voltammetry (CV) and differential pulse voltammetry (DPV). Measurements were conducted using a potentiostat/galvanostat (Gamry Reference 3000 P/G system). In this three-electrode system, Pt wire, Ag/AgCl electrode, and Pt-Co@rGO modified GCE was used as a counter, reference, and working electrode, respectively.

### Synthesis of GO

A modified Hummer’s method was used to obtain GO from graphite. In the first step of the synthesis, 2 g of powder graphite, 55 mL of 96.4% sulfuric acid (H_2_SO_4_), and 1 g of sodium nitrate (NaNO_3_) were mixed, and the mixture was put into an ice bath for one hour. 7.5 g of KMnO_4_ (potassium permanganate) was gradually added to the mixture in an ice bath. During these operations, the temperature was kept below 5 °C. The mixture was then removed from the ice bath and blended for 2 hours. During the process, the temperature was maintained within the range of 30–50 °C. At the final stage of the synthesis process, 250 mL of distilled water was slowly added to the mixture and stirred for one hour. Then, 4.4 mL of hydrogen peroxide (35.7%) added, and mixed for 2 hours at 35–40 °C. At this stage, the colour of the mixture turned from black to brown. The mixture was washed with purified water (pH = 7) and filtered. After filtration, the remaining material was dried in the oven at 60 °C for one day^31^.

### Synthesis of Pt-Co@rGO

To prepare Pt-Co@rGO nanocomposite, rGO was prepared with the help of GO and hydrazine hydrate. Then, Pt-Co loading was performed using a microwave-assisted reduction method. Pt-Co@rGO (1:1) nanocomposite was obtained as follows: 20 mg of rGO (1: 1) was stirred in 30 mL of ethylene glycol by ultrasonic treatment for 1 hour. Then 0.025 mmol of PtCl_4_ and CoCl_2_ solution were added and dissolved using a magnetic stirrer. NaOH was added for adjusting the pH of the solution. The obtained solution was placed into the microwave the oven (1000 W, 2.45 GHz) and heated for 5 minutes, and repeated eight times for 30 seconds. The solution was then centrifuged, washed with deionized water, followed by drying in a vacuum oven.

## Results and Discussion

### Characterization of the morphology and structure of Pt-Co@rGO

**Figure 2:**
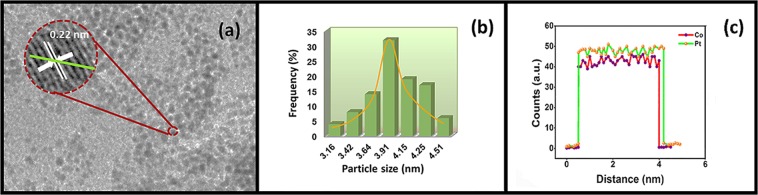
(**a**) TEM/HR-TEM image (**b**) particle size histogram and (**c**) EELS line profile of Pt-Co@rGO.

HRTEM and TEM were used to detect the particle size distributions, atomic lattice fringes and the morphology of Pt-Co@rGO. TEM/HRTEM images showed that most of the particles were spherical, and no agglomeration was detected, as shown in Fig. 2(a). The prepared bimetallic nanoparticles were uniformly dispersed over rGO surface, and atomic lattice fringe of 0.22 nm was calculated with the help of HRTEM image, which confirms the alloy formation when compared to the nominal Pt value of 0.23 nm^32–34^. Besides, as shown in Fig. 2(b), the average particle size was found to be 3.91 ± 0.44 nm. TEM/EELS image indicates that both Pt and Co are co-existed in the prepared composite (Fig. 2(c)), which confirms the alloy formation through the green line in Fig. 2(a).

**Figure 3:**
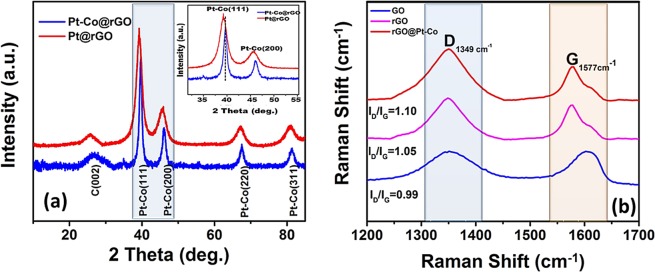
(**a**) XRD and (**b**) Raman spectrum of Pt-Co@rGO nanocomposites.

The XRD pattern of Pt-Co@rGO is shown in Fig. 3(a). The diffraction peaks at about 2θ = 40.09°, 46.44°, 67.92°, and 81.76° originate from the planes Pt-Co (111), (200), (220) and (311) related to face-centred cubic (fcc) crystal framework. 2θ = 24.50° diffraction peak corresponded to the mixture of reduced GO. All the diffraction patterns for the Pt-Co@rGO are shifted slightly to the higher 2θ values, which indicate the lattice contraction because of the substitution of Co into Pt. This case also confirms the alloy formation in prepared nanocomposites. Besides, using the equation (1), the lattice parameter (*α*Pt) value calculated as 3.910 Å, which is very close to 3.923 Å for pure Pt^35,36^, and mean crystallite size of the metal particles was calculated as 3.77 ± 0.71 nm, using equation (2) ^29^.

1 $$\mathrm{Sin}\,{\rm{\theta }}=\frac{{\rm{\lambda }}\sqrt{{h}^{2}+{k}^{2}+{l}^{2}}}{2a}({\rm{for}}\,{\rm{a}}\,{\rm{cubic}}\,{\rm{structure}})$$

2 $$d(\AA )=\frac{k\lambda }{\beta \,\cos \,\theta }$$

Where k is the constant (0.9), λ is the wavelength (1.54056 Ǻ) of the X-ray, β is the maximum half of the full width of the respective breaking peak (rad), θ is angle at the maximum peak (rad) position.

Raman spectroscopy is an effective method for distinguishing the regular and irregular structure of carbon in materials. The Raman spectra of GO, rGO, and Pt-Co@ rGO are shown in Fig. 3(b). The I_D_/I_G_ ratios for GO, rGO, and Pt-Co@rGO were found to be 0.99, 1.05, and 1.10, respectively. Increasing I_D_/I_G_ ratios clearly show the functionalization of the graphene oxide surface.

**Figure 4:**
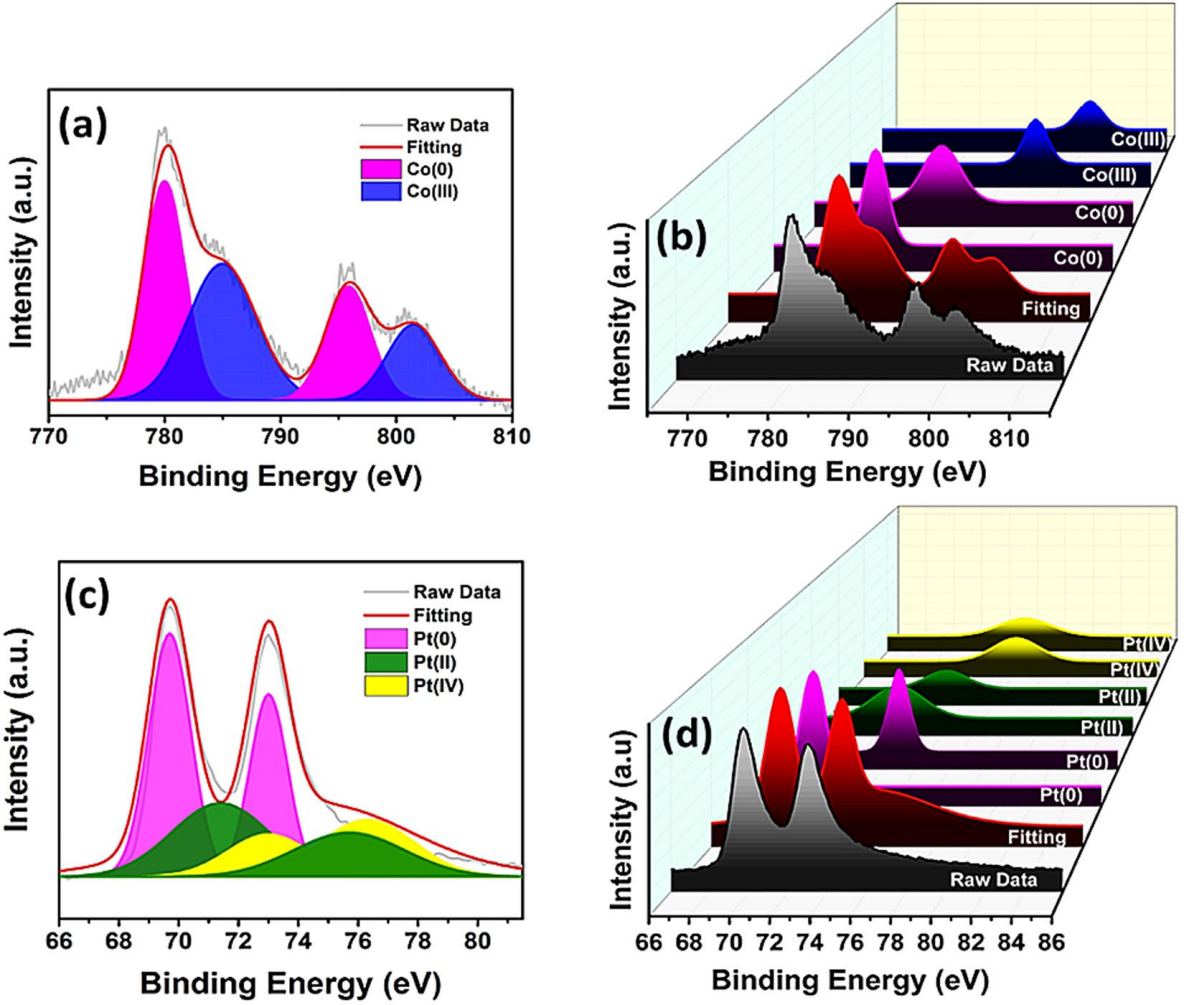
(**a**) Co 2p XPS spectrum and (**b**) its 3D view. (**c**) Pt 4 f XPS spectrum and (**d**) its 3D view.

The oxidation states and chemical compositions of Pt-Co elements in Pt-Co@rGO nanocomposites were examined by XPS. The Co-2p and Pt-4f spectra were analyzed using Gauss-Lorentzian functions, and Shirley-shaped background correction was applied. The amount of the species was evaluated by calculating the integration areas of each peak. In XPS spectrum, the correct binding energies (0.3 eV) were determined by reference to the C 1 s peak at 284.6 eV. The XPS spectra, given in Fig. 4(a), indicated that the surface of the prepared composite was mostly metallic. The experimental binding energies were compared with the literature, for cobalt binding energy, the changing of 2p_3/2_ peak to lower energy indicated the alloying of Cobalt with Platinum^37^. The results showed that during the production of the nanocomposite, Pt (0) and Co (0) are mostly available in Pt-Co@rGO, instead of oxidized compounds. The reason for the higher oxidation state of Pt and Co showed in Fig. 4 may be caused by chemical oxidation or surface oxidation by environmental oxygen during the preparation process. Since the susceptibility factor of Pt-4f is 3–4 times larger than Co-2p, the peak region of Pt is greater than Co.

### Verifying the scan rate

After the characterization studies, Pt-Co@rGO based biosensor was employed for the detection of ascorbic acid (AA), dopamine (DA) and uric acid (UA). For this purpose, the effect of the scan rate for the measurements was investigated. Figure S1a–c shows the oxidation peaks of the UA, DA, AA at different scan rates. As shown in these figures, the peaks are directly proportional to the increasing scan rate. Since there was a diffusion-controlled process for the complete realization of the reaction, the gradual shift of the oxidation peak potentials to the positive values was observed. This indicates that the modified electrode had a kinetic limit in the reaction between the redox regions and biomolecules. As a summary, the reactions of ascorbic acid (AA), dopamine (DA) and uric acid (UA) are diffusion-controlled processes.

### Effect of pH on electrochemical responses

It is very important to optimize experimental parameters to achieve precise and selective results. The effect of the pH on the separation of CV responses was studied with phosphate buffer, at pH range from 3.0 to 9.0, in 2.5 × 10^−3^ M of DA. Figure S2 shows that the anodic and cathodic peak intensities of DA changed negatively, and the anodic and cathodic peak current intensities decreased as the pH increased (*poly*-DOPA formation). The current densities against pH were linear within the range of pH of 3.0 to 9.0 (Fig. S1(b)). These figures also indicate that the amounts of electrons and protons in electrocatalytic oxidation were determined in equal amounts. As a summary, a maximum anodic current response was obtained at pH 3.0 for DA. The optimum pH value was determined as 3.0 for further studies to ensure the simultaneous determination of AA, DA, and UA.

### Electrochemical behavior of AA, DA, and UA in modified electrodes

**Figure 5:**
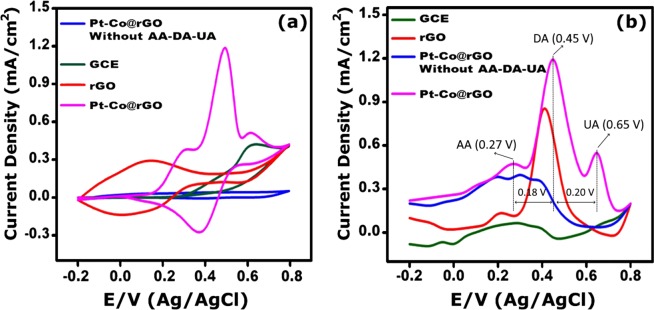
(**a**) CV results of modified electrodes for AA, DA, and UA (each 4 × 10^−3^ M, scan rate 50 mV s^−1^) and (**b**) related DPV curves (each 4 × 10^−3^ M, DPV: −0.20 to + 0.80 V, pH of 3.0, 0.1 M PBS).

Differential Pulse Voltammetry (DPV) and Cyclic Voltammetry (CV) measurements were performed to obtain electrochemical behaviour (sensing efficiency and performance) of modified electrodes for AA, DA, and UA. The CV and DPV data of the electrodes (bare GCE, rGO, Pt-Co@rGO) is shown in Fig. 5 for the simultaneous determination of AA, DA, and UA in 0.1 M PBS. Although the DPV graphs of Pt-Co@rGO were similar to rGO/GCE, the peak currents increased significantly (Fig. 5(b)). Oxidative peak potentials of AA, DA, and UA was found to be 0.27 V, 0.45 V, 0.65 V, respectively. As marked in Fig. 5(b), potential differences of 0.18 V and 0.20 V were achieved between AA-DA and DA-UA, respectively. This results support that the oxidative potential peaks are well separated and the sensitive simultaneous detection is allowed for these biomolecules.

### Individual and simultaneous detection of AA, DA, and UA using Pt-Co@rGO

**Figure 6:**
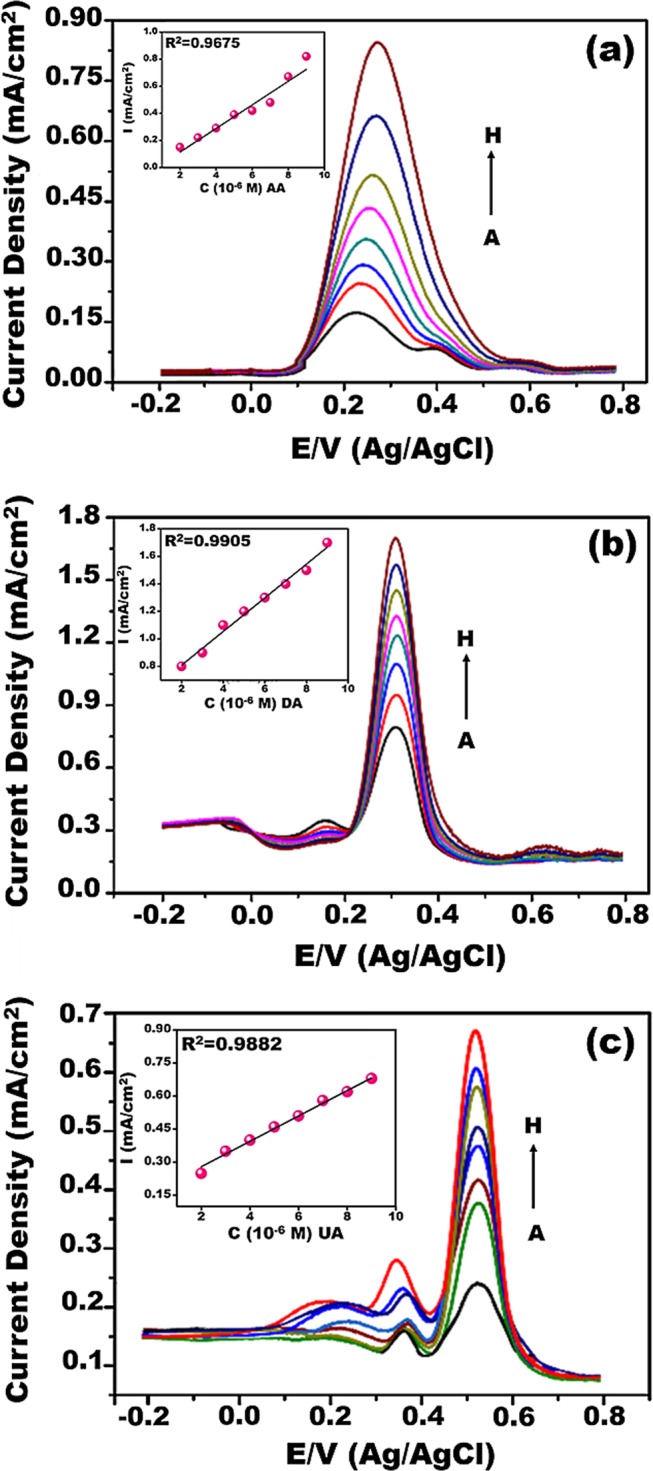
DPV profiles of Pt-Co@rGO for (**a**) different concenterations of AA in the presence of 1 × 10^−3^ M UA and DA (A: 2 × 10^−3^, B: 3 × 10^−3^, C: 4 × 10^−3^, D: 5 × 10^−3^, E: 6 × 10^−3^, F: 7 × 10^−3^, G: 8 × 10^−3^, and H: 9 × 10^−3^ M), (**b**) different concenterations DA in the presence of 1 × 10^−3^ M AA and UA (A: 2 × 10^−3^, B: 2.5 × 10^−3^, C: 3 × 10^−3^, D: 3.5 × 10^−3^, E: 4 × 10^−3^, F: 4.5 × 10^−3^, G: 5 × 10^−3^, and H: 5.5 × 10^−3^ M to H), (**c**) different concenterations of UA in the precence of 1 × 10^−3^ M DA and AA. (A: 1 × 10^−3^, B: 2 × 10^−3^, C: 3 × 10^−3^, D: 4 × 10^−3^, E: 5 × 10^−3^, F: 5.5 × 10^−3^, G: 6 × 10^−3^, and H: 7 × 10^−3^ M). Insets are the related calibration graphs.

Individual detection of AA, DA, and UA was performed under optimum conditions in PBS at pH 3.0, in a potential range of −0.20 to 0.80 V. Experiments were conducted based on the principle of keeping the concentration of two species constant, while the concentration of the target molecule is being changed. Figure 6 shows that DPV curves of different target biomolecule concentrations in the coexistence of 1 × 10^−3^ M each of other biomolecules. In Fig. 6(a) electrochemical oxidation peak current of AA was proportionally increased with the increasing concentration of AA. However, the peak currents of DA and UA stayed constant. Similar way, when the DA concentrations were altered in the presence of AA and UA, only the DA oxidation peak current was increased that corresponded to increasing DA concentrations (Fig. 6(b)). Figure 6(c) demonstrate the DPV curves of various UA concentrations in the presence of AA and DA, the oxidation peak current of UA was increased linearly with the increasing amount of UA. In this case, a slight increase was also observed on the peak currents of AA and DA, but it is not a significant affection compared to UA. The square of the correlation coefficients (R^2^) were calculated as 0.9675, 0.9905, and 0.9882 for AA, DA, and UA, respectively. In the lights of these results, it can be concluded that these biomolecules have very good linear measuring range, do not interfere each other, and can be utilized for simultaneous detection AA, DA, and UA with good selectivity. Compared to the GCE; the presence of functional groups, metal nanocomposites, smaller particle size and higher active surface area of Pt-Co@rGO/GCE electrode increased the current density and allowed the well-defined oxidative peaks for AA, DA, and UA.

**Figure 7:**
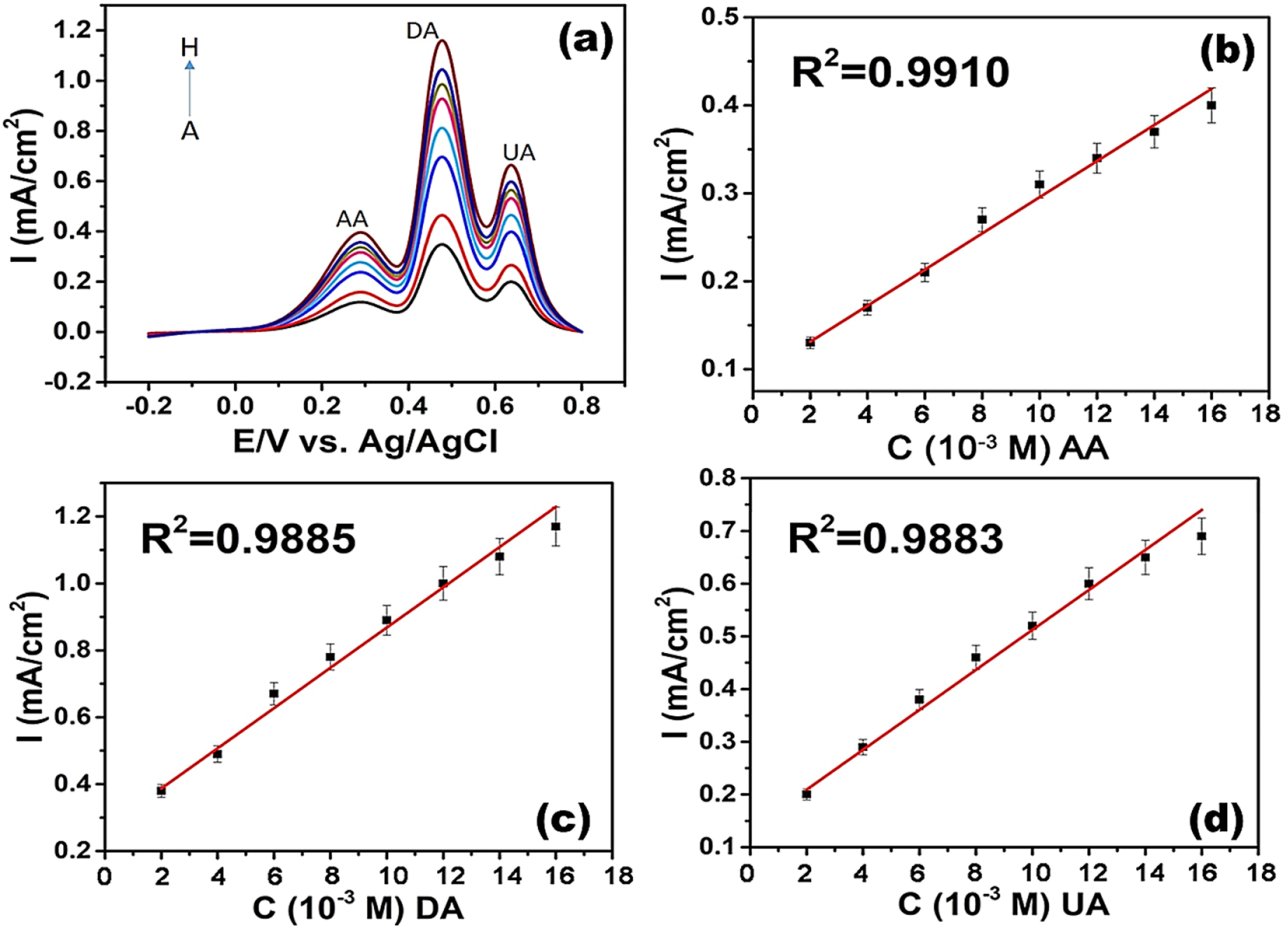
DPV profiles of (**a**) Pt-Co@rGO in 0.1 M PBS buffer (pH 3.0) containing various concentrations of AA, DA, UA. (A-H) From bottom to top: 2 × 10^−3^ to 9 × 10^−3^ M for AA; from 2 × 10^−3^ to 5 × 10^−3^ M for DA; from 1 × 10^−3^ to 7 × 10^−3^ M for UA, respectively. The related calibration graphs for (**b**) AA, (**c**) DA, and (**d**) UA.

The simultaneous detection of AA, DA, and UA was performed under optimum conditions in the PBS in a potential range of −0.20 to 0.80 V. During the experiment, the concentrations of all three analytes were changed and very good responses were obtained. Figure 7(a–d) show the DPV profiles of Pt-Co@rGO in 0.1 M PBS buffer (pH 3.0) containing various concentrations of AA, DA, UA, and the related calibration graphs. The linear range and detection limit of Pt-Co@rGO was calculated for each analyte, as listed in Table S1. The linear range of Pt-Co@rGO was found to be 170–200; 35–1500 and 5–800 µM for AA, DA, and UA, respectively. The detection limit of the prepared composite was calculated as 0.345; 0.051; 0.172 µM for AA, DA, and UA, respectively. Table S1 gives the comparison of previously reported electrochemical sensors for simultaneous determination of AA, DA, and UA. As shown in Table S1, our result indicates that very high sensitivity and efficiency was achieved on the electrochemical detection of UA, DA, and AA. The stability and durability of prepared biosensor were also studied. For this purpose, the prepared electrode kept for more than ten weeks and tested in the same conditions. It was seen that the modified electrode remained stable with very good sensitivity.

### Effect of repeated voltammetric cycle

Since many carbon-based electrodes were susceptible to rapid fouling, the prepared Pt-Co@rGO composite was examined for contamination. Generally, DA electro-oxidation results in rapid contamination of the electrode. Therefore, repetitive cycling for the electro-oxidation of 2.5 × 10^−3^ M DA in 0.1 M PBS at pH 3.0 & 7.0 was performed by using Pt-Co@rGO and bare GCE (Fig. S3). The results revealed that there is a fouling effect at pH 7.0; however, no-contamination was observed at pH 3.0. The reason for this phenomenon is that electro-oxidation of DA at neutral or basic pH which leads to the development of *poly*-DOPA on the surface of the electrode. This cause a decrease in the sensitivity by repetitive cyclic scans.

The effects of electro-oxidation of AA and UA (in combination and individually) on the susceptibility to dopamine detection using repetitive CVs and DPVs in PBS medium (pH 3.0) and optimized conditions were also investigated. Figure S4a–c shows the repeated CVs for the electro-oxidation of DA, both individually and together, in AA, UA concentrations of 4 × 10^−3^ M. The change on the current signals was examined by the respective DPV profiles under optimized conditions (Fig. S4d). It can be concluded that increasing number of scans slightly affects the electro-oxidation of DA, and the sensitivity of AA and UA^27,38,39^.

### Real sample applications

Application of Pt-Co@rGO based biosensor in real samples was achieved in PBS medium. Samples were analysed with the standard addition method. For this purpose, AA, DA, and UA were added to the diluted (100 times) serum samples as standard solution and recovery studies were performed. The results were given in Table 1. The recoveries obtained from the serum samples at three different concentrations were in a range of 96–101% which indicates there is no significant interference. As a result, the prepared Pt-Co@rGO sensor can be used in routine tests of serum samples for simultaneous detection of AA, DA, and UA in micromolar levels, and suitable for the applications in daily use.

**Table 1:** The results of the analysis of the serum examples (200 µL of the serum example with three different concentrations of AA, DA, UA (I, II & III). DPV conditions: 180 s; 0.1 M PBS solution (pH of 3.0); potential range −0.20 to + 0.80 V (UDL: under detection limit).

Serum sample	Found (10^−6^ M)			Spiked (10^−6^ M)			Recovery (10^−6^ M)			% Recovery		
	AA	DA	UA	AA	DA	UA	AA	DA	UA	AA	DA	UA
I	UDL	UDL	33.0	100	10	10	96 ± 1.2	9.7 ± 0.9	42.8 ± 0.3	96	97	99.5
II	UDL	UDL	34.0	250	25	50	252 ± 0.4	24.6 ± 0.3	84.5 ± 0.2	100.8	98.4	100.6
III	UDL	UDL	33.0	500	50	100	505 ± 0.3	49.9 ± 0.3	132.9 ± 0.9	101.0	99.8	99.9

## Conclusions

The characteristics of Pt-Co@rGO biosensor, such as enhanced sensor sensitivity, linear calibration range, detection limit, etc. were studied, and compared with previously reported ECSs in the literature. The developed Pt-Co@rGO sensor showed relatively better detection limits and had a wider linear range compared to the available ECSs in use. The perfect electro-sensing activity of the Pt-Co@rGO enabled the simultaneous determination of AA, DA, and UA (LOD = 0.345 µM, 0.051 µM, 0.172 µM, respectively). Moreover, the improved electrode had better sensitivity, selectivity, and precision (1.98, 1.72, and 2.3% for AA, DA, and UA, respectively). Pt-Co@rGO biosensor was exhibited great stability, even it was kept under dry conditions for more than ten weeks. In conclusion, the study highlights future developments for the individual and simultaneous determination of AA, DA, and UA in the presence of other biomolecules.

## Supplementary information

SUPPLEMENTARY INFO
