# Supplementary material for: Composites of Bimetallic Platinum-Cobalt Alloy Nanoparticles and Reduced Graphene Oxide for Electrochemical Determination of Ascorbic Acid, Dopamine, and Uric Acid
Source: Sci Rep. 2019 Aug 22;9:12258. doi: 10.1038/s41598-019-48802-0 (PMC6706408; doi:10.1038/s41598-019-48802-0)
Supplement: Supplementary file 1 — SUPPLEMENTARY INFO [file 41598_2019_48802_MOESM1_ESM.docx]

**Supporting information**

**Composites of Bimetallic Platinum Cobalt Alloy Nanoparticles and Reduced Graphene Oxide for Electrochemical Determination of Ascorbic Acid, Dopamine, and Uric Acid**

Buse Demirkan^a^, Sait Bozkurt^a^, Aysun Şavk^a^, Kemal Cellat^a^, Fulya Gülbağca^a^, Mehmet Salih Nas^a,b*^, Mehmet Hakkı Alma^b^ and Fatih Sen*^a^

^a^Sen Research Group, Department of Biochemistry, Faculty of Arts and Science, Dumlupinar University, Evliya Çelebi Campus, 43100 Kütahya, Turkey.

^b^Department of Environmental Engineering, Faculty of Engineering, University of Igdir, Igdir, Turkey.

^*^Corresponding author: fatih.sen@dpu.edu.tr, mehmet.salih.nas@igdir.edu.tr.

Tel:90 274 265 20 31 -37 02 Fax:90 274 265 20 56

**Electrochemical studies**

AA, DA, UA solutions were put into an electrochemical cell including 0.1 M phosphate buffer (pH 3.0) and three electrodes were placed. The potential interval was selected from −0.20 to +0.80 V and the current evaluation was performed by CV and DPV measurements. The operating conditions as follow: scan rate of 50 mV/s, pulse width of 0.1 s, and pulse amplitude of 2 mV. The concentrations of AA, DA, and UA were determined simultaneously by evaluating the oxidation peak currents.

**Preparation of electrodes**

Glassy carbon (GC) electrodes, 3 mm in diameter, polished with 0.05 mm alumina to a mirror-finish prior to experiments. In order to prepare modified electrodes, typically, 3 mg of nanocomposite was added into 0.5 mL 0.05 wt. % Nafion solutions, and then the mixture was treated for 1 h with ultra-sonication for uniform dispersion. A measured volume (30 mL) of this mixture was dropped by a microsyringe onto the surface of the GC electrode. Pt-Co@rGO modified-GC electrode was employed as a working electrode in our experiments.


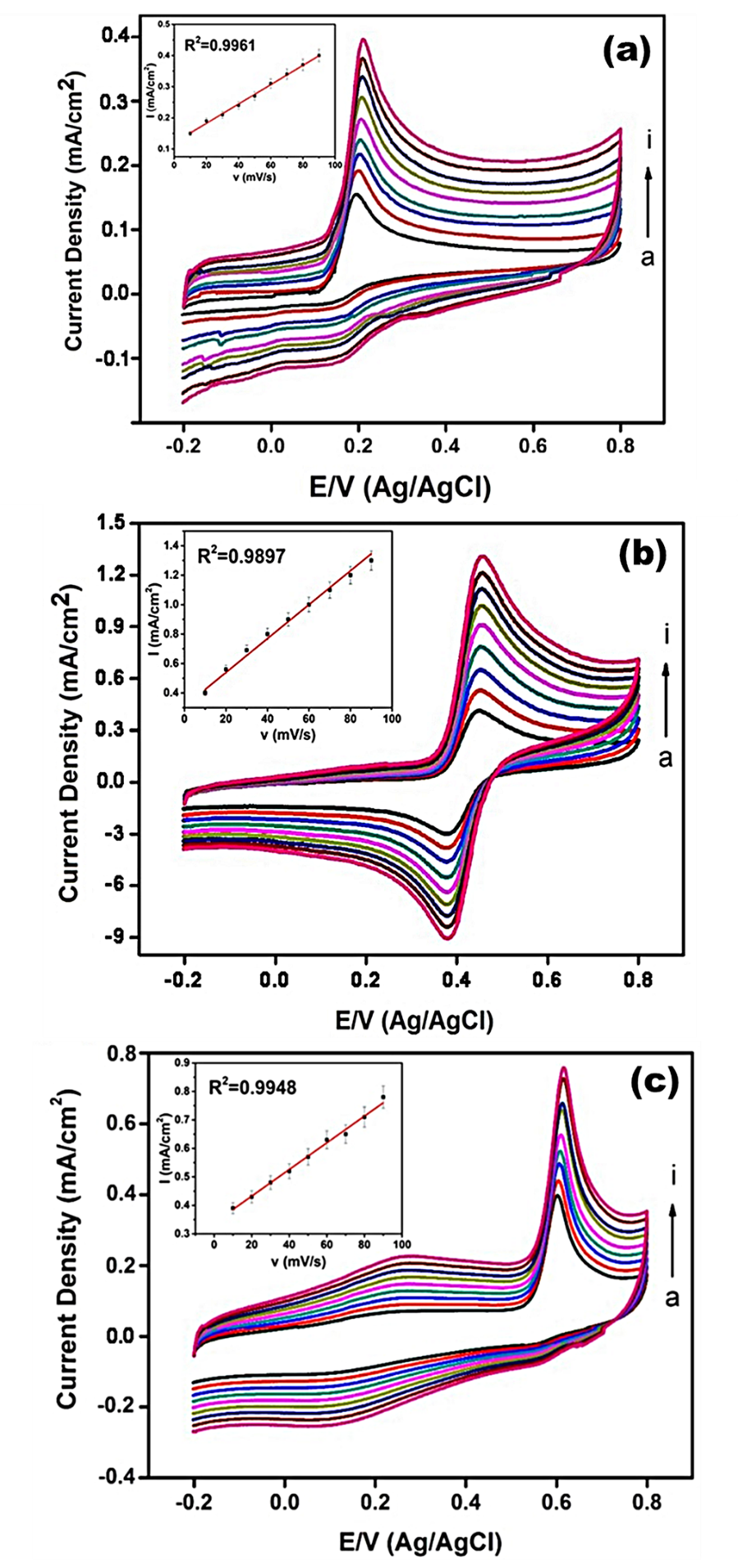


**Fig. S1.** Cyclic voltammograms of 4×10^-3^ M of (a) AA, (b) DA, and (c) UA at various scan rates (from 10 to 90 mV s^-1^). Insets: corresponding I vs υ plots.

**
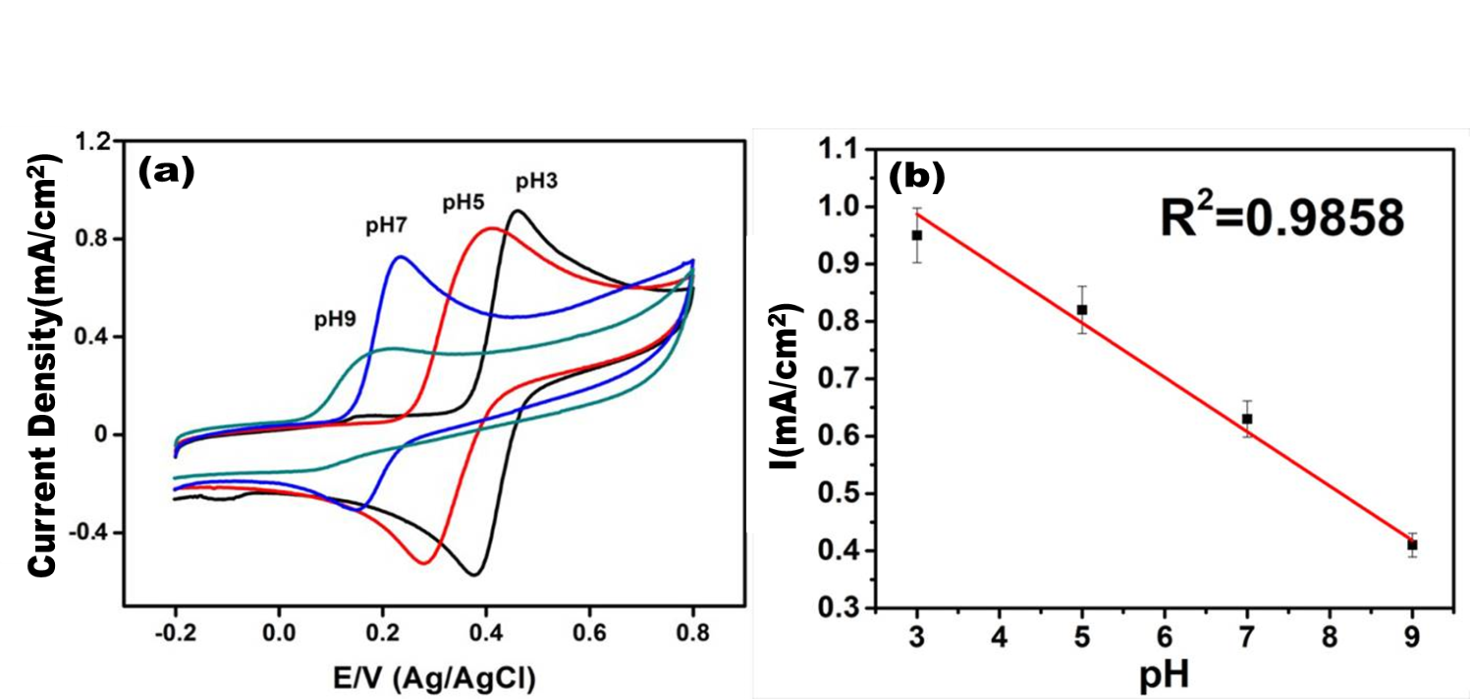
**

**Fig. S2.** CVs of the Pt-Co@rGO /GCE (a) after addition of 2.5×10-3 M DA at different pH’s (3.0, 5.0, 7.0, and 9.0) (b) plots of the anodic current vs. pH’s (3.0, 5.0, 7.0, and 9.0).

**
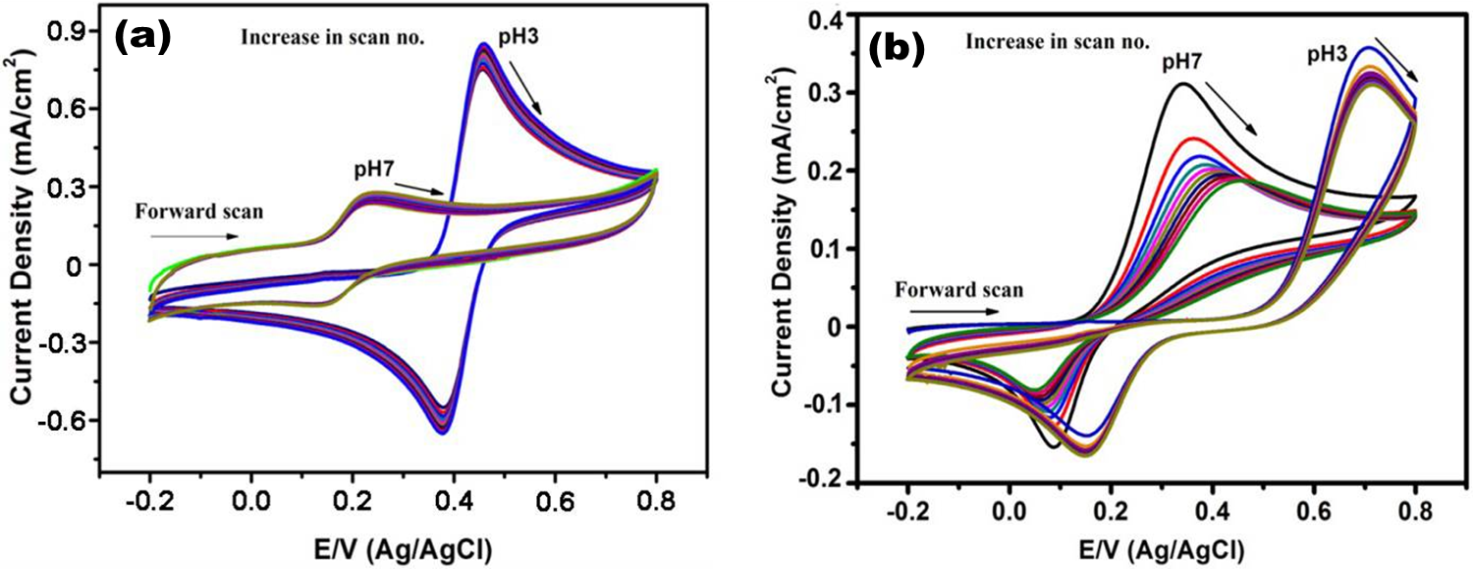
**

**Fig. S3.** Repetitive cycling for the electro-oxidation of 2.5×10^-3^M DA in 0.1 M phosphate buffer at pH 3.0 and 7.0 **(a)** Pt-Co@rGO /GCE **(b)** bare GCE.

**
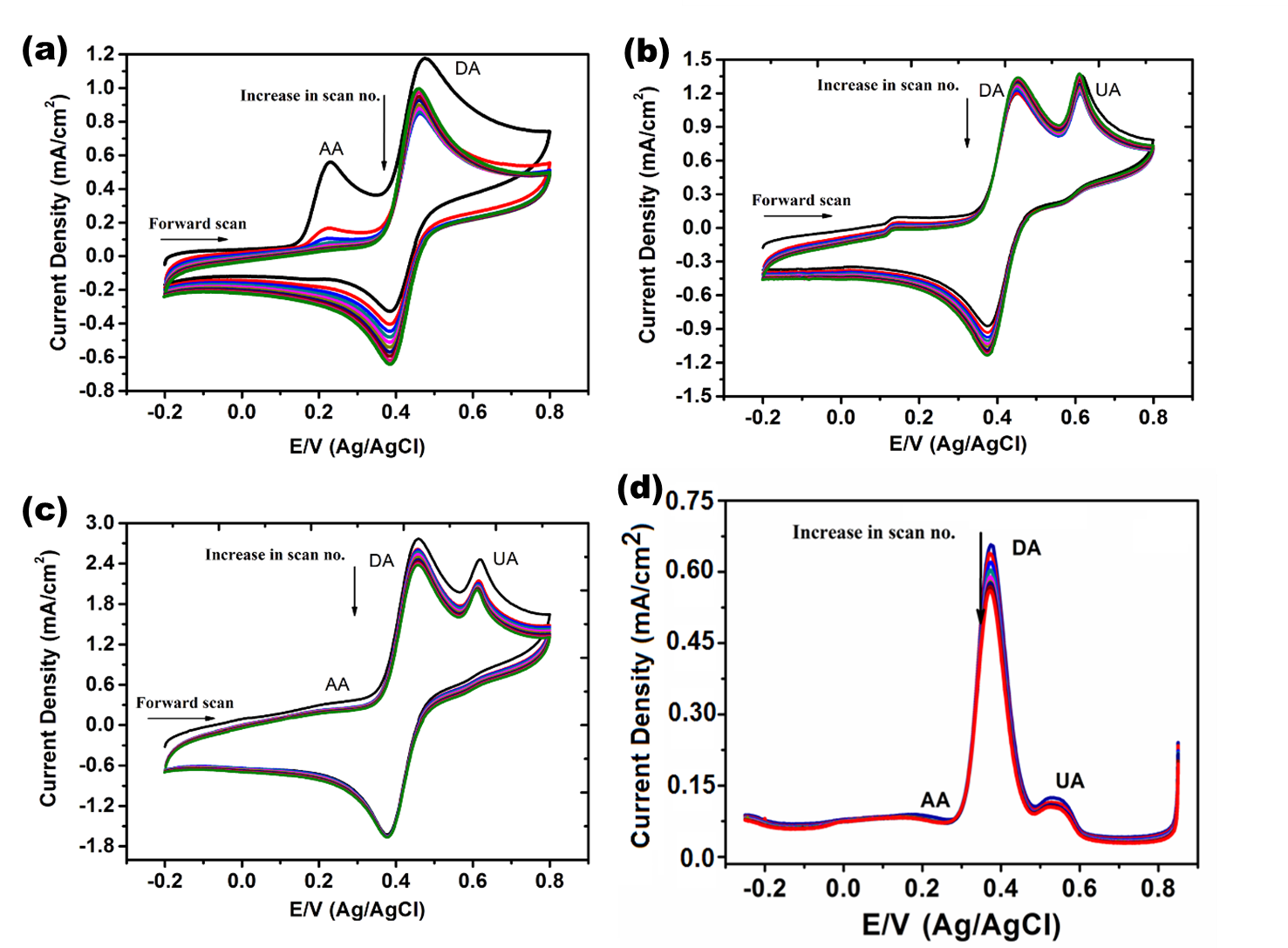
**

**Fig. S4.** Repetitive cycling for the simultaneous electro-oxidation of (a) 4×10^-3^ M of AA and DA (b) 4×10^-3^ M of DA and UA (c) 4×10^-3^ M of AA, DA, and UA in phosphate buffer at pH of 3.0, and (d) corresponding DPV profile

**Table S1.** Comparison of previously reported electrochemical sensors data for simultaneous determination of AA, DA, and UA.

| Electrode | Linear range (10-6M) | | | Detection Limit (10-6M) | | | References |
| --- | --- | --- | --- | --- | --- | --- | --- |
|  | **AA** | **DA** | **UA** | **AA** | **DA** | **UA** |  |
| PoPD/E-RGOa | - | 10-800 | - | - | 7.5 | - | 1 |
| e-FGPEb | 20-400 | 0.5-35 | 0.5-35 | 2 | 0.01 | 0.02 | 2 |
| GEF/CFEc | 45.4-1483.2 | 0.7-45.21 | 3.78-183.87 | 24.7 | 0.5 | 2 | 3 |
| CTABGO/MWNTd | 5-300 | 5-500 | 3.0-60 | 1.0 | 1.5 | 1.0 | 4 |
| PImox-GOe | 75-2275 | 12-278 | 3.6-249.6 | 18 | 0.63 | 0.59 | 5 |
| IL-G/GCEf | - | 5-275 | 1-400 | - | 0.812 | 0.513 | 6 |
| Pt/RGO | - | 10-170 | 10-130 | - | 0.25 | 0.45 | 7 |
| (Fe3O4-NH2)@ (GS)g | 5-1600 | 0.2-38 | 1.0-850 | 0.074 | 0.126 | 0.056 | 8 |
| ERGOh | 500-2000 | 0.5-60 | 0.5-60 | 250 | 0.5 | 0.5 | 9 |
| SDS- MWCNTs/GCE | 400-3500 | 0.8-80 | 4-30 | 3 | 0.01 | 0.04 | 10 |
| RGOi | - | - | 0.1-10 | - | - | 0.05 | 11 |
| PdNPs/GR/CS/GC Ej | 100-4000 | 0.5-200 | 0.5-200 | 20 | 0.1 | 0.17 | 12 |
| PEDOT-modified  Ni/Si MCP | 20-1400 | 12-48 | 36-216 | 10 | 1.5 | 2.7 | 13 |
| Methoxypolyethyle ne glycols/GCE | - | 1-140 | - | - | 0.0468 | - | 14 |
| CPE/GNS k | - | 2-1000 | - | - | 0.85 | - | 15 |
| TNCs1-GCE | 80-1400 | 0.4-60 | 10-70 | 14±0.56 | 0.28±0  .02 | 1.6±0.0  5 | 16 |
| AuNPs@MIPs | - | 0.02-.54 | - | - | 0.0078 | - | 17 |
| PPy-RGOl | - | 0.06-8 | - | - | 0.006 | - | 18 |
| BPPG/MWCNT/H ^m^ | 40-280 | 0.01-0.07 | 2-14 | 1.94 | 0.003 | 0.1 | 19 |
| SGN/NiPcn | 25-1050 | 0.25-10 | 5.0-175 | 0.12 | 0.08 | 0.22 | 20 |
| N-PCNPso | 80-2000 | 0.5-30 | 4-50 | 0.74 | 0.01 | 0.02 | 21 |
| Nano-Cu-PSAIII/GCEp | 0.30-730 | 0.02-65 | 0.25-107 | 0.15 | 0.01 | 0.10 | 22 |
| Poly(tyrosine)/MW CNT q | 50-1000 | 0.1-30 | 1-350 | 2.0 | 0.02 | 0.30 | 23 |
| Modified GCE | 25-300 | 3-300 | 5-70 | 23.38 | 2.67 | 4.70 | 24 |
| P-4-ABA/GCE r | 20-800 | 5.0-100 | 1.0-80 | 5.0 | 1.0 | 0.5 | 25 |
| DpAu/PTCA- Cyst/GCE s | 20-700 | 2-402 | 0.40-252 | 6.40 | 0.67 | 0.12 | 26 |
| Graphene modified electrode | - | 2.5-100 | - | - | 0.5 | - | 27 |
| AuNWt | - | 0.2-600 | - | - | 0.026 | - | 28 |
| NG u | 5-1300 | 0.5-170 | 0.1-20 | 2.2 | 0.25 | 0.045 | 29 |
| PVP/Graphene | - | 5×10-4-  1.13×103 | - | - | 0.002 | - | 30 |
| Cysteamine- Functionalized MWCNT | - | 0.2-100 | 1-100 | - | 0.02 | 0.1 | 31 |
| Pre-treated GCE | - | 0.1-12 | - | - | 0.03 | - | 32 |
| N-CNRs v | - | 0.008-15 | - | - | 0.009 | - | 33 |
| Graphene | - | - | 2-120 | - | - | 0.6 | 34 |
| Pt-Co@rGO -GCE | 170-2000 | 35-1500 | 5-800 | 0.345 | 0.051 | 0.172 | Present work |

^a^ poly(o-phenylenediamine) (PoPD)/E-RGO hybrid composite,

^b^ Exfoliated flexible graphite paper,

^c^ Graphene flowers/Carbon fiber,

^d^ CTAB functionalizedgrapheneoxide/multiwalledcarbon nanotube composite,

^e^ Overoxidizedpolyimidazole and grapheneoxide,

^f^  Ionic liquid functionalized graphene,

^g^ amino-group functionalized mesoporous Fe_3_O_4_@graphene sheets,

^h^ electrochemically reduced graphene oxide,

^I^ reduced grapheneoxide,

^j^ Palladium nanoparticle/graphene/chitosan/glassy carbon electrode,

^k^ Carbon paste electrode modified with graphene nanosheet,

^l^ polypyrrole-reduced graphite oxide core–shell microspheres,

^m^ basal plane pyrolytic graphite (BPPG) electrode modified with 1,4-naphthoquinone (NQ)adsorbed on multiwalled carbon nanotubes (MWCNT),

^n^ Nickel(II) phthalocyanine on mesoporous SiO_2_/C carbon ceramic matrices,

^o^ Nitrogen doped porous carbon nanopolyhedra,

^p^ Cu nanoparticles (nano-Cu)–poly(sulfonazo III) (PSA III) modified glassy carbon electrode,

^q^ poly (tyrosine)/carboxyl functionalized multi-walled carbon nanotubes composite film,

^r^ A polymerized film of 4-aminobutyric acid on the surface of glassy carbon electrode,

^s^ Deposited gold nanocrystals enhanced porous PTCA–Cys layer,

^t^ Gold nanowire modified,

^u^ Nitrogen doped graphene,

^v^ Nitrogen-doped carbon nanorods.

**Reference**

1. X. Liu, H. Zhua and X. Yang, RSC Adv., 2014, 4, 3706.
2. W. Caia, T. Laia, H. Dub and J. Ye, Sens. and Actuators B, 2014, 193, 492.
3. J. Du, R. Yue, F. Ren, Z. Yao, F. Jiang, P. Yang and Y. Du, Biosens. Bioelectron., 2014,

53, 220.

1. Y. J. Yang and W. Li, Biosens. Bioelectron., 2014, 56, 300.
2. X. Liu, L. Zhang, S. Wei, S. Chen, X. Ou and Q. Lu, Biosen. Bioelectron., 2014, 57,

232.

1. C. Wang, P. Xu, and K. Zhuo, Electroanalysis, 2014, 26, 191.
2. T. Xu, Q. Zhang, J. Zheng, Z. Lv, J. Wei, A. Wang and J. Feng, Electrochim. Acta,

2014, 115, 109.

1. D. Wu, Y. Li, Y. Zhang, P. Wang, Q. Wei and B. Du, Electrochim. Acta, 2014, 116, 244.
2. L. Yang, D. Liu, J. Huang and T. You, Sens. and Actuators B, 2014, 193, 166.
3. J. Zhang, Z. Zhu, J. Zhu, K. Li and S. Hua, Int. J. Electrochem. Sci., 2014, 9, 1264.
4. Z. Zhang and J. Yin, Electrochim. Acta, 2014, 119, 32.
5. X. Wang, M. Wu, W. Tang, Y. Zhu, L. Wang, Q. Wang, P. He and Y. Fang, J.

Electroanal. Chem., 2013, 695, 10.

1. S. Yu, C. Luo, L. Wang, H. Peng and Z. Zhu, Analyst, 2013, 138, 1149.
2. Y. Wu, L. Cui, Y. Liu, G. Lv, T. Pu, D. Liu and X. He, Analyst, 2013, 138, 1204.
3. M. Bagherzadeh and M. Heydari, Analyst, 2013, 138, 6044.
4. S. Zhou, H. Shi, X. Feng, K. Xue and W. Song, Biosen. Bioelectron., 2013, 42, 163.
5. C. Xue, Q. Han, Y. Wang, J. Wu, T. Wen, R. Wang, J. Hong, X. Zhou and H. Jiang,

Biosens. Bioelectron., 2013, 49,199.

1. T. Qian, S. Wu and J. Shenab, Chem. Comm., 2013, 49, 4610.
2. A. X. Oliveira, S. M. Silva, F. R. F. Leite, L. T. Kubota, F. S. Damos and R. C. S. Luz,

Electroanalysis, 2013, 25, 723.

1. S. B. A. Barros, A. Rahim, A. A. Tanaka, L. T. Arenas, R. Landers and Y. Gushikem,

Electrochim. Acta, 2013, 87, 140.

1. P. Gai, H. Zhang, Y. Zhang, W. Liu, G. Zhu, X. Zhang and J. Chen, J. Mater. Chem. B,

2013, 1, 2742.

1. L. Zhang, W. J. Yuan and B. Q. Hou, J. Electroanal. Chem., 2013, 689, 135.
2. Y. Wang and C. Bi, Journal of Molecular Liquids, 2013, 177, 26.
3. Z. Temocin, Sens. and Actuators B, 2013, 176, 796.
4. X. Zheng, X. Zhou, X. Ji, R. Lin and W. Lin, Sens. and Actuators B, 2013, 178, 359.
5. W. Zhang, Y. Chai, R. Yuan, J. Han and S. Chen, Sens. and Actuators B, 2013, 183, 157.
6. X. Ma, M. Chao and Z. Wang, Anal. Methods, 2012, 4, 1687.
7. M. S. Hsu, Y. L. Chen, C. Y. Lee, and H. T. Chiu, ACS Appl. Mater. Interfaces, 2012, 4,

5570.

1. Z. H. Sheng, X. Q. Zheng, J. Y. Xu, W. J. Bao, F. B. Wang, X. H. Xia, Biosens.

Bioelectron., 2012, 34, 125.

1. Q. Liu, X. Zhu, Z. Huo, X. He, Y. Liang and M. Xu, Talanta, 2012, 97, 557.
2. S. Shahrokhian, A. M. Shakib, M. Ghalkhani and R. Saberi, Electroanalysis, 2012, 24,

425.

1. D. Q. Huang, C. Chen, Y. M. Wu, H. Zhang, L. Q. Sheng, H. J. Xu and Z. D. Liu, Int. J.

Electrochem. Sci., 2012, 7, 5510.

1. D. Yuan, X. Yuan, S. Zhou, W. Zou and T. Zhou, RSC Adv., 2012, 2, 8157.
2. M. Chao, X. Ma and X. Li, Int. J. Electrochem. Sci., 2012, 7, 2201.
